# Ultrasonic Enhancement of Chondrogenesis in Mesenchymal Stem Cells by Bolt-Clamped Langevin Transducers

**DOI:** 10.3390/mi14010202

**Published:** 2023-01-13

**Authors:** Jinhyuk Kim, Hyuncheol Bae, Hyuk-Soo Han, Jungwoo Lee

**Affiliations:** 1 Department of Electronic Engineering, Kwangwoon University, Seoul 01897, Republic of Korea; 2 Department of Orthopedic Surgery, Seoul National University College of Medicine, Seoul 03080, Republic of Korea

**Keywords:** mesenchymal stem cells, chondrogenesis, Langevin transducers

## Abstract

We recently investigated the design and fabrication of Langevin-type transducers for therapeutic ultrasound. Effect of ultrasonic energy arising from the transducer on biological tissue was examined. In this study, the transducer was set to radiate acoustic energy to mesenchymal stem cells (MSCs) for inducing differentiation into cartilage tissue. The average chondrogenic ratio in area was 20.82% in the control group, for which no external stimulation was given. Shear stress was applied to MSCs as the contrast group, which resulted in 42.66% on average with a 25.92% minimum rate; acoustic pressure from the flat tip causing transient cavitation enhanced chondrogenesis up to 52.96%. For the round tip excited by 20 V_pp_, the maximum differentiation value of 69.43% was found, since it delivered relatively high acoustic pressure to MSCs. Hence, the results from this study indicate that ultrasound pressure at the kPa level can enhance MSC chondrogenesis compared to the tens of kHz range by Langevin transducers.

## 1. Introduction

High-intensity focused ultrasound (HIFU) transducers have widely been used to produce various effects, such as thermal necrosis and hyperthermia. Conventional HIFU transducers were modeled to make single or multiple focal points that lead to thermal effects on target regions, e.g., uterine fibroids [1] and breast cancer [2], and low intensity pulsed ultrasound (LIPUS) was engineered to treat those diseases and diabetes [3] and bone fractures [4]. In general, both transducers have been built in a stacked form and composed of a piezoelectric element, backing layer, and matching layer for effective energy transfer at a few MHz. In order to allow sufficient energy for practical purposes, however, ultrasound at tens of kHz is preferred, due to greater depth of penetration and wider acoustic exposure. In our recent works [5,6], bolt-clamped transducers, also known as Langevin transducers, were fabricated to radiate 40 kHz ultrasound in therapeutic use. Their structures include a pre-stress bolt, metal masses, piezoelectric stacks, and an acoustic rod with a small tip. In particular, we conducted an experimental study where biological specimens such as porcine fat tissue and bovine muscle were subjected to acoustic pressure from the 2.9 mm tip, and demonstrated that the ultrasound had a thermal effect on those tissues [7].

It is well-known that mesenchymal stem cells (MSCs) can differentiate into various tissues—adipose, cartilage, etc. Due to its anatomical and structural complexity, in fact, articular cartilage is one of the most challenging tissues to treat for clinical applications. Several studies have examined the impact of mechanical stress on in vitro development into cartilaginous tissue in light of the fact that chondrogenesis was effectively induced by biomechanical force in human joints [8,9]. For example, it was reported that shear and compressive force could induce the chondrogenic differentiation of MSCs [10]. LIPUS at the MHz frequency range was used to enhance MSCs’ chondrogenesis [11]. Although the effect was generally promising, it was often seen that no significant change was observed in comparison with the control group. In a LIPUS stimulation, in particular, the transducer position should be precisely manipulated to locate the focal point, and the attenuation proportional to its driving frequency cannot be negligible. Since traditional stacked-type transducers with low frequency ranges were extremely bulky and additional acoustic coupling gel was needed, it was hard to adapt such transducers into an in vitro stimulation environment with MSCs.

In this study, Langevin transducers applied 40 kHz ultrasound to human MSCs to investigate whether our devised transducer affects their chondrogenic process through mechanical stimuli. To this end, the acoustic pressure emitted from the transducer was measured with hydrophone system, and the occurrence of transient cavitation was analyzed through Fast Fourier Transform (FFT). Further testing was conducted with a digital orbital shaker to compare the effects of simple mechanical shear stress and longitudinal oscillation from our transducer. Three transducers equipped with flat and round tips were set to directly transmit acoustic energy to those cells. After three-week culturing, cells were evaluated for the effectiveness of chondrogenic induction by ultrasonic stimulation. For quantitative analysis, the relative ratio of cartilage grown area to that of stained MSCs were computed by ImageJ.

## 2. Materials and Methods

### 2.1. MSCs Isolation and Culture

In this experimental study, synovium tissues were obtained from five female osteoarthritis patients (age 66 to 72 years) undergoing total knee arthroplasty. In all patients, the Kellgren Lawrence grade was 4, and osteoarthritis had progressed on the medial side of the knee. Synovium was harvested from the suprapatellar pouch. Ethical approval for this study was obtained from Seoul National University Hospital Institutional Review Board. Those who had inflammatory arthritis, prior knee joint infection, and intraarticular trauma were excluded. Synovial tissue was minced in phosphate-buffered saline and digested with 0.02% collagenase (Sigma, St. Louis, MO, USA) overnight. Cells were filtered from undigested tissue with 70 μm sieves and centrifuged at 1500 rpm for 5 min. Then, cells were cultured in low glucose Dulbecco’s modified eagle’s medium (LG-DMEM, Gibco, Paisley, Scotland, UK) with 10% fetal bovine serum and 1% penicillin/streptomycin/amphotericin at 37 °C with 5% CO_2_. The medium was changed after 48 h, and nonadherent cells were removed during this procedure. We used early-passage synovium-derived stem cells (SDSCs) (P2) in this study.

### 2.2. Chondrogenesis of MSCs

The 5 × 10^5^ SDSCs were centrifuged at 1500 rpm for 5 min to obtain cell pellets. Cell pellets were cultured in chondrogenic medium (LG-DMEM) containing 0.1 mmol/L ascorbic acid 2-phosphate, 100 nmol dexamethasone, 40 g/ mL proline, 100 U/mL penicillin, 100 g/mL streptomycin, and ITS Premix (BD Biosciences, Franklin Lakes, NJ, USA) supplemented with transforming growth factor beta 1 (TGF-ß1). SDSCs pellets were allowed to differentiate for up to 21 days. Medium was refreshed every 3–4 days.

### 2.3. Histology

For histological evaluation of glycosaminoglycan (GAG) synthesis, cell pellets from each group were stained with Safranin-O and fast green staining at day 21. Staining was performed as described in our previous study [12]. Cell pellets were harvested, fixed in 4% paraformaldehyde for 4 h at room temperature, embedded in Tissue Tek embedding medium (Sakura Finetek, Torrance, CA, USA), and incubated for 20 min at −70 °C. Pellets were sectioned 5 mm thickness at −20 °C and mounted on slides. The sections were air-dried and post-fixed at −20 °C with acetone for 15 min.

### 2.4. Langevin Transducer

**Figure 1:**
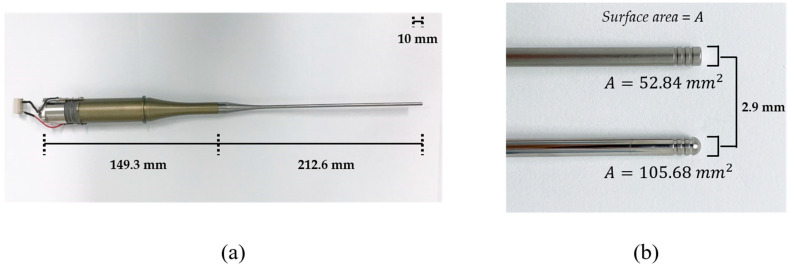
(**a**) Assembled Langevin transducer. (**b**) Tip shapes: (**top**) flat tip, (**bottom**) round tip.

The transducer was composed of back mass, two pairs of piezoelectric rings, front mass, and thin attachable rod, all of which were clamped by a central metal bolt. Stainless steel (SUS304) was selected as the bolt due to its low thermal conductivity and high mechanical durability. Since SUS 304 has higher acoustic impedance than the piezoelectric layer, it was also used as a back mass that reflects back the ultrasonic energy from piezoelectric elements towards the forward direction. Four rings of hard-type PZT-4 (C-203, Fuji Ceramic Corporation, Fujinomiya, Japan) were employed because of its high mechanical quality factor, Q_m_. As a front mass, aluminum (Al7075) that has cost-effective and ease of manufacturing is anodized to prevent corrosion from external factors. In order to amplify ultrasonic energy, an exponential-shaped acoustic booster was additionally built in the front mass, where the diameter ratio of two end surfaces determines the magnifying factor. These structures are benefits of Langevin transducer for transferring high-power acoustic energy to media. Titanium was processed to fabricate attachable thin rods coupled with acoustic boosters. The rod tips were shaped to be either flat or round. The diameter of the rod tips was 2.9 mm, and the radiating area of the spherical tip was twice that of the flat one. The whole transducer and enlarged tips are shown in Figure 1.

### 2.5. Measurement of Acoustic Pressure

**Figure 2:**
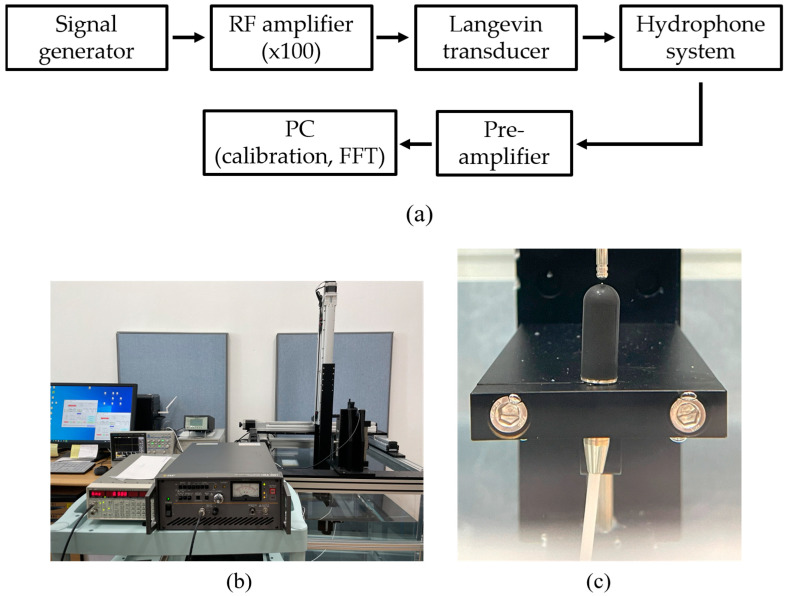
Pressure calibration system: (**a**) flow chart of measurement procedure, (**b**) complete system, (**c**) enlarged view of the transducer with a round tip and hydrophone in the water tank.

Acoustic pressure emitted from the transducer’s tip is characterized by a calibration system that consists of a signal generator (SG382, Stanford Research System, Sunnyvale, CA, USA), RF amplifier (HAS 4051, NF, Yokohama, Japan), water tank, 3-axis motorized positioner; and a manual stage with a hydrophone (8103, Bruel & Kjaer, Naerum, Denmark), pre-amplifier (2692-0S1, Bruel & Kjaer, Naerum, Denmark), and personal computer (PC). Sinusoidal waveforms from the generator with amplitudes 100 mV_peak-to-peak_ (V_pp_), 200 mV_pp_, and 400 mV_pp_ are augmented by the amplifier with a gain of 100. A hydrophone in the water tank measured the acoustic pressure emitted by the immersed tip of the excited transducer. The pre-amplifier sent the RF signals to the PC for calibration with a resolution of 100 μv/Pa. Figure 2 shows the experimental setup and enlarged views of immersed spherical tip and hydrophone sensor.

### 2.6. Experimental Setup

**Figure 3:**
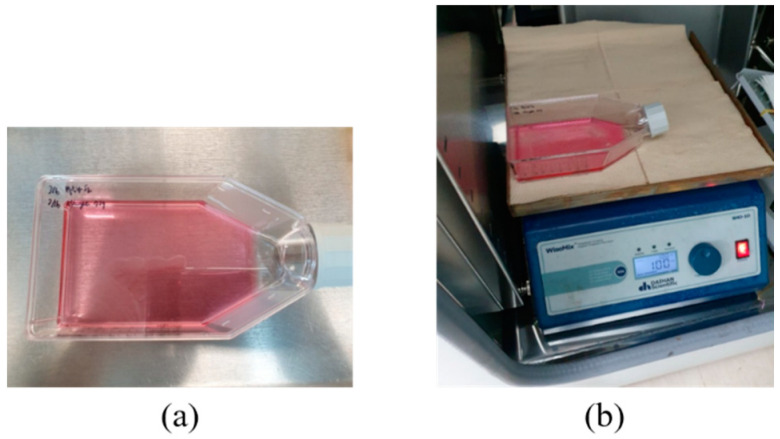
Shear stress test: (**a**) T flask containing MSCs, (**b**) digital shaker for shear stress.

**Figure 4:**
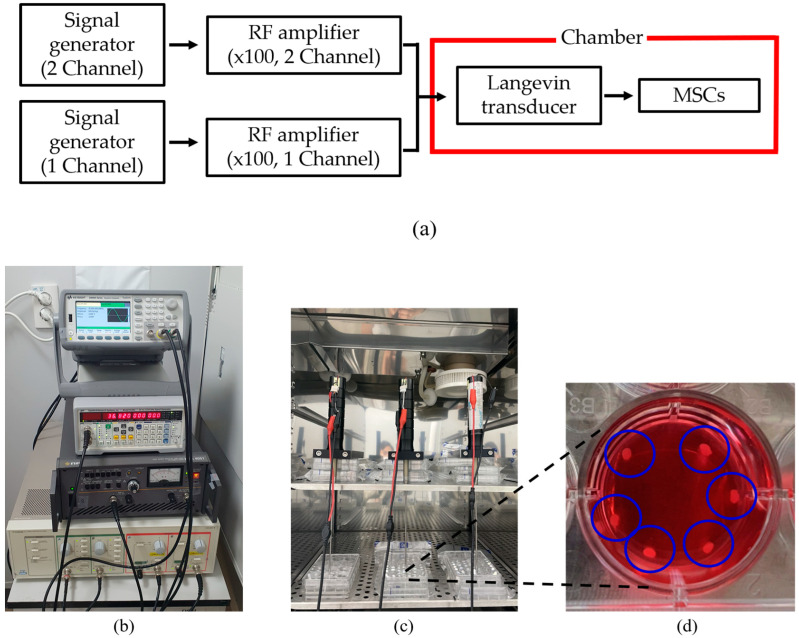
Configuration for ultrasound test: (**a**) flow chart of equipment operation, (**b**) system for transducer excitation, (**c**) test chamber, (**d**) enlarged view of liquid badge and MSCs inside blue circles.

A digital orbital shaker (SHO-1D, DAIHAN Scientific, Wonju, Korea) was arranged to apply external mechanical force to the MSCs in T flask in a constant temperature and humidity chamber (3131, Thermofisher Scientific, Waltham, MA, USA). Among various external forces, shear stress could be applied to stem cells in order for them to differentiate into cartilage tissue using our shaker machine over 24 h. Furthermore, three Langevin transducers were set to radiate acoustic energy into the medium. They were clamped by a customized jig. The liquid medium was filled in a dish of 24 well plates, and the diameter of each well was 22.7 mm. Continuous sinusoidal wave for transducer excitation was produced from 2-channel signal generator (33600A series, KEYSIGHT, Santa Rosa, CA, USA) and augmented by a multi-channel amplifier (7068, Yokogawa, Musashino, Japan) with a gain of 100. Note that such excited transducers emit acoustic energy from 6 pm to 9 am for three weeks. To protect transducers from mechanical fatigue and electrical malfunction, devices were not excited between 9 am and 6 pm. To fix the titanium rod, a hole was punched in the center of the dish’s cover. The tip was set to 5 mm away from the well’s bottom, and MSCs were placed along the well’s edge to protect them from direct acoustic exposure. Both shaker and ultrasonic test settings are represented in Figure 3 and Figure 4, respectively.

## 3. Results and Discussion

**Figure 5:**
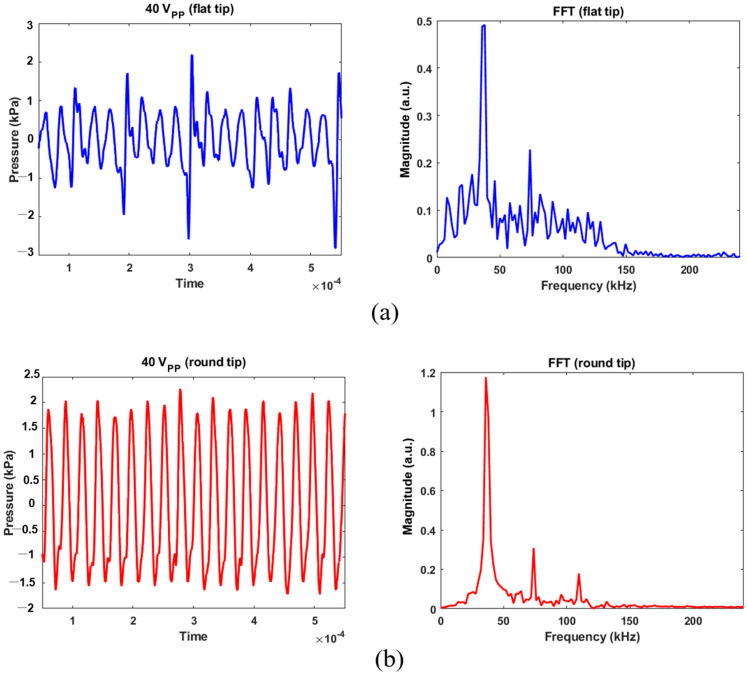
Acoustic pressure calibration for 40 V_pp_ excitation and its FFT results: (**a**) flat tip, (**b**) round tip.

**Figure 6:**
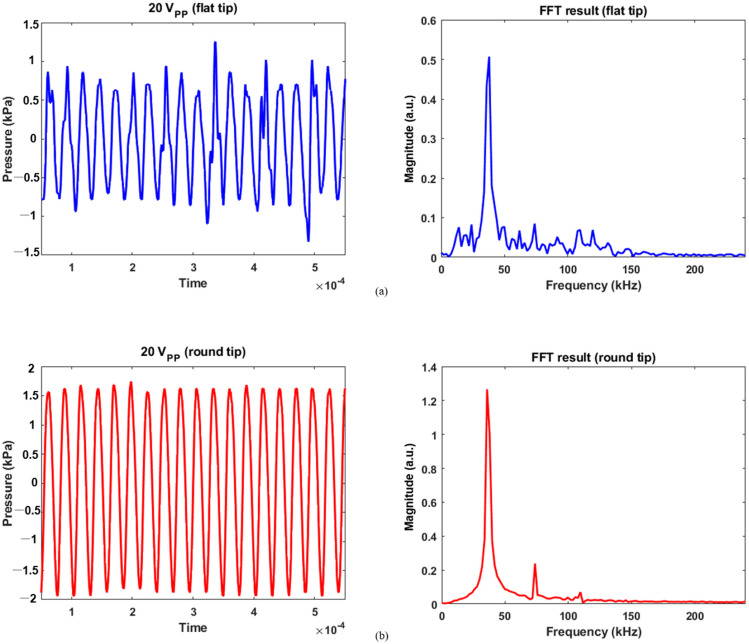
Calibration data for 20 V_pp_ and its FFT results: (**a**) flat tip, (**b**) round tip.

Calibrated acoustic pressure data are shown in Figure 5 and Figure 6. The hydrophone sensor was located at 5 mm from the transducer’s tip, taking into account the distance between the transducer’s tip and the bottom of the experimental dish. The AC voltage applied to the transducer was controlled at 10, 20, or 40 V_pp_ to prevent physical damage to MSCs. A Fast Fourier Transform (FFT) was used to characterize bubble’s behavior from acoustic cavitation. For 40 V_pp_ in Figure 5a, the maximum positive and negative pressures were 2.19 and −2.81 kPa for the flat tip. It was found that the magnitude was 0.5 at the fundamental frequency (=37.84 kHz) and 0.23 at the second harmonic (=73.71 kHz). Wideband noise was observed up to 250 kHz, which indicates that transient cavitation was caused by high-intensity ultrasound [13]. Figure 5b revealed that maximum and minimum pressures of 2.27 and −1.71 kPa were obtained for the round tip, respectively. Despite few noise components being observed in the given frequency range, there were three distinct peaks of 1.18, 0.31, and 0.18 at the fundamental and harmonic frequencies.

For 20 V_pp_ excitation, the flat tip generated 1.25 and −1.33 kPa as maximum positive and negative pressures in Figure 6a, respectively. As reduced electrical energy was input, the recorded pressure contained a lower level of noise over the same frequency range. The magnitude was 0.51 for the fundamental frequency and 0.09 for the second harmonic. Similarly, the pressure was measured between 1.75 and −1.94 kPa for round tip. As compared with Figure 5b, Figure 6b showed that noise components were more suppressed; their FFT results were 1.27, 0.24, and 0.07 at the fundamental, second, and third harmonic frequencies. This implies that as the area of round tip was greater than that of flat radiator, the bubble collapse was less significant. A previous study also confirmed this fact: when a tip has smaller active area for acoustic emission, the bubble oscillation becomes more violent than that of a round tip [14]. When a spherical tip generates radial wave propagation, it induces a unique phase of acoustic streaming not seen for the flat tip, resulting in a large cavitation gas volume. In contrast, a rectangular tip causes a different fluid flow distribution, which produces bubble collapse activity at the tip’s surface. Furthermore, the cavitation phenomenon’s shielding effect renders acoustic waves to lose energy while penetrating the cavitation zone. It causes pressure plots to fluctuate, as seen in the flat-tip results [15,16]. The resultant values were determined by averaging the measured acoustic power from five separate trials, as summarized in Table 1.

**Figure 7:**
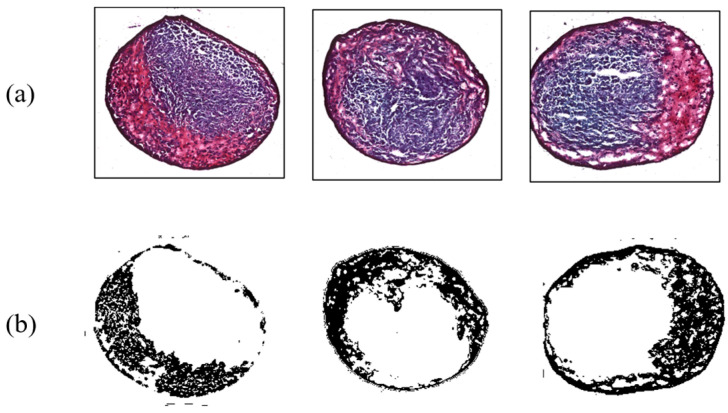
Chondrogenesis results for the control group. (**a**) Safranin-O-stained MSCs; (**b**) chondrogenic portion identified by ImageJ.

**Figure 8:**
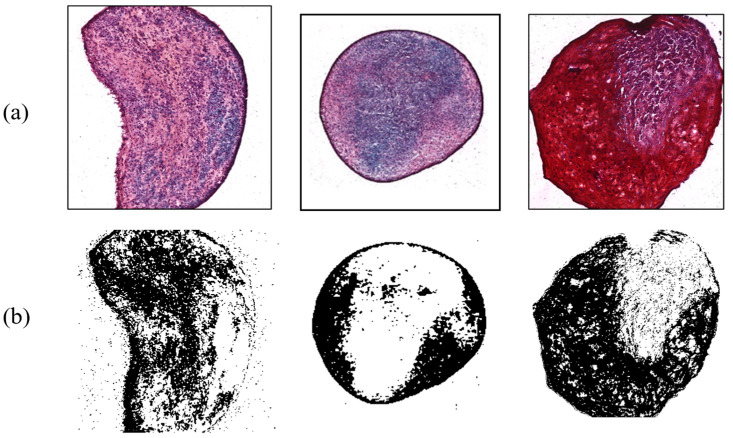
Chondrogenesis with shear force. (**a**) Safranin-O-stained MSCs; (**b**) ImageJ results.

**Figure 9:**
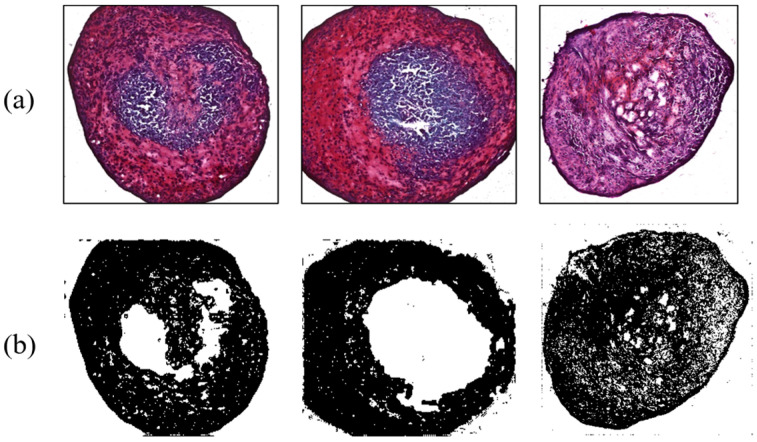
Ultrasonic effect on chondrogenesis for the flat tip. (**a**) Safranin-O-stained MSCs; (**b**) ImageJ results.

**Figure 10:**
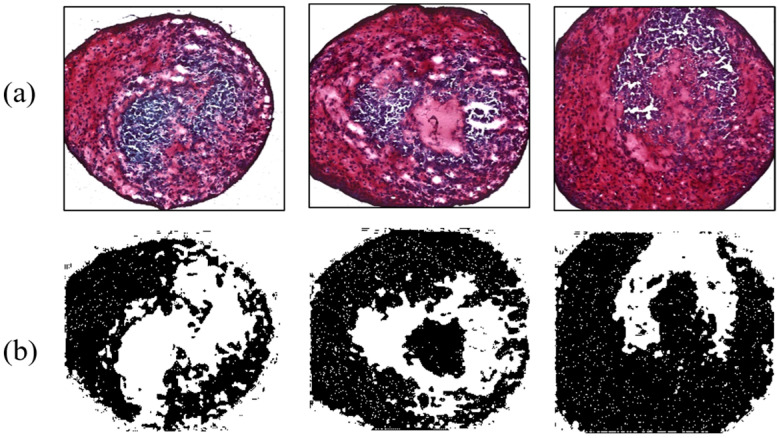
Enhanced chondrogenesis for the round tip. (**a**) Safranin-O-stained MSCs; (**b**) ImageJ results.

In addition, results for MSC differentiation into chondrocytes are presented in Figure 7, Figure 8, Figure 9 and Figure 10. Safranin-O-stained cells observed via microscope were analyzed by ImageJ to calculate the chondrogenic area ratio in MSCs. Note that the stained part of chondrogenesis appears red–purple through the microscope, and ImageJ converts it to black, and the necrosis portion, which is shown in blue–purple, was converted to white. The number of control groups in the MSC pellets was nine, and they were cultured after three weeks of growth in the chamber. Among them, three typical cells are shown as the control group without external stimulus in Figure 7. Computed differentiation ratios were 18.17%, 20.67%, and 24.71%—left to right. The maximum ratio was 35.01%; the total averaged chondrogenesis ratio was 20.82%.

In contrast, shear force arising from the digital shaker was applied to MSCs for comparing the results from the control group. During the three-week period, they were cultured and influenced by the force for 15 h a day. There were six target MSCs, with an average ratio of 42.66%. Figure 8 exhibits typical examples of shear force results with chondrogenesis percentages of 34.38%, 25.92%, and 47.46%, respectively. This indicated that the differentiation ratio was generally higher than that of control group. Shear stress, a type of biomechanical stimulus for chondrocytes, is critical to allowing synovial fluid to nourish cartilage tissue, carrying waste material, and keeping chondrocytes metabolically active via a diffusion and fluid-convection mechanism [17]. According to a previous study, it was found that shear stress increased chondrogenesis by analyzing the amount of GAG synthesis [18].

As another contrast group, MSCs influenced by 38 kHz ultrasound were analyzed to evaluate the effect of acoustic stimulation. Four MSCs were interrogated by a flat-tip transducer activated by 40 V_pp_. Acoustic energy from the tip enhanced chondrogenesis, causing maximum and minimum ratios of 61.23% and 40.42%, respectively; the average value was 52.96%. The flat tip and 20 V_pp_ resulted in ratios of 47.3% and 55.2%. Chondrogenic results with the flat tip were always higher than those of the control group. Meanwhile, the round tip excited by 10, 20, and 40 V_pp_ affected 10 MSCs. Among them, cartilage tissue influenced by 20 V_pp_ showed 69.43% differentiation, the highest value in all contrast group throughout the experiment. As a minimum rate was 41.19%, the round tip always produced higher differentiation than the control group. The averaged ratios under the electrical conditions were 52.49%, 52.83%, and 50.85%. Figure 9 and Figure 10 present chondrogenic results induced by ultrasound from flat and round tips, respectively. Test results of all groups are summarized in Table 2.

As the round tip radiated higher acoustic energy than the flat tip, the maximum differentiation was induced accordingly. As compression and rarefaction phases alternate, it can periodically apply mechanical stress to MSCs. Compression can lead to an internal increase in hydrostatic pressure which benefits differentiation into cartilage tissue [19].

Moreover, the chondrogenic process requires calcium ions (Ca^2+^) to synthesize a matrix component. Entry of Ca^2+^ is controlled by voltage-operated calcium channels, transient receptor potential channels, and so on [18]. It is possible to infer that the acoustic waves in this study had an impact on chondrogenesis through modulating Ca^2+^ channels, based on research demonstrating that ultrasound may alter the ion channel [20,21]. Cell membrane permeability could be increased by this intense cavitation [22,23]. The shock wave caused a transient disruption of cell membrane, allowing for easier transport of extracellular materials. Furthermore, such strong waves could produce membrane strain and displacement between cells and its surroundings, which provide an increase in permeability of cell membrane [24,25]. Despite the frequent use of focused ultrasound, non-focused transducers have also taken advantage of cavitation for sculpting body contour with low-intensity ultrasound, where plasma free-fatty acid and norepinephrine in extracellular fluid around the perirenal adipose tissue of rat abdomens were increased by mobilizing fat through enhanced norepinephrine secretion after a ten-minute acoustic application [26]. Increasing cell permeability may be crucial because diffusion and fluid convection mechanisms affect chondrogenesis. Even with lower pressure than a round tip, a flat tip could have a greater chondrogenic ratio on average by resulting in more transient cavitation effect, as previously discussed.

**Figure 11:**
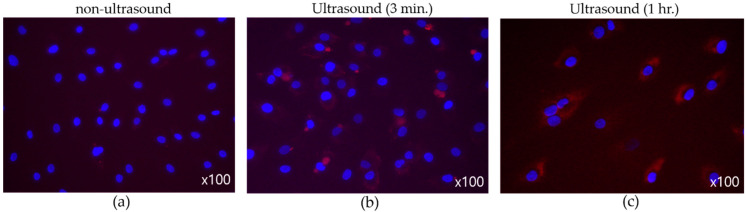
(Flat tip, 40 V_pp_) Effect of ultrasound on membrane permeability. (**a**) No sonification, (**b**) three-minute exposure, (**c**) one-hour exposure.

Figure 11 suggests the effect of ultrasound on cell-membrane permeability, where synovial cells were the target and the nuclei were dyed to reveal blue spots. Substances permeated into cell membrane were stained in red. To induce the cavitation effect, transducers equipped with flat tip were driven with 40 V_pp_. Infused materials were hardly seen in those cells in Figure 11a. After three-minute ultrasonic application (Figure 11b), however, surroundings of nuclei were tinged with red due to the increase in cell permeability. When ultrasonic exposure was prolonged to one hour, in particular, more extensive fluorescent cells were found in Figure 11c, which indicates reinforced material intake across the membrane.

## 4. Conclusions

In this study, chondrogenesis of MSCs by 38 kHz ultrasound from Langevin transducers was investigated. A test without using ultrasonic stimuli was executed as a control during three weeks in a specially designed chamber. In contrast, shear force was applied to MSCs by a digital orbital shaker for the same period. As compared with control group of 20.82%, mechanical stress enhanced cartilage-tissue differentiation with an averaged ratio of 42.66%. In the presence of ultrasound, a flat tip generating transient cavitation induced greater chondrogenesis on average, whereas a round tip emitting higher acoustic pressure causes more differentiation into chondrocytes. Through pressure calibration with hydrophone and FFT analysis, more evident transient cavitation was also discovered in the case of the flat tip, which demonstrates that the cavitation effect led to an increase in cell-membrane permeability. This may be because acoustic pressure transferred compressional energy to MSCs for enhancing hydrostatic force and stimulated the activation of the ion channel. Therefore, it was found that ultrasound might give rise to more effective differentiation into cartilage tissue than simple shear stress force. Consequently, the results from this work suggest the effectiveness of ultrasound radiation from Langevin transducers on in vitro chondrogenesis in MSCs. In our future work, we will design precise and accurate setups to find enhancement of chondrogenesis in vivo because all experiments in this study were performed in vitro. To accomplish this, we developed a small transducer with a low frequency range and characterize acoustic parameters, such as penetration depth and area of focal point. We will investigate the impact of low-frequency ultrasound on the differentiation of MSCs in cartilage tissue in small animal joints.

## Figures and Tables

**Table 1:** List of pressure calibration based on tip shape.

Resultant Values		10 V_pp_	20 V_pp_	40 V_pp_
Averaged maximum pressure (kPa)	Flat tip	- ^1^	1.16	2.19
	Round tip	1.20	1.66	2.30
Magnitude at fundamental frequency (a.u.)	Flat tip	- ^1^	0.48	0.53
	Round tip	0.98	1.15	1.26

^1^ Pressure data are not available for the flat tip, since they are too low for hydrophone.

**Table 2:** Summary of chondrogenesis ratios in control and contrast groups.

	Maximum (%)	Minimum (%)	Averaged (%)
Control	33.36	5.48	20.82
Shear force	58.03	25.92	42.66
Flat tip (40 V_pp_)	61.23	40.42	52.96
Round tip (20 V_pp_)	69.43	41.19	52.83

## Data Availability

Experiment data are available on request to Jungwoo Lee.
